# First successful case of chemical pleurodesis with oxytetracycline through Rocket® Pleural Vent™: ambulatory pneumothorax device for pneumothorax

**DOI:** 10.1002/rcr2.471

**Published:** 2019-08-07

**Authors:** Wang Chun Kwok, Ka Chun Cheng, David Chi Leung Lam, Terence Chi Chun Tam

**Affiliations:** ^1^ Department of Medicine Queen Mary Hospital Hong Kong Hong Kong

**Keywords:** Chemical pleurodesis, Pleural Vent™, pneumothorax

## Abstract

A 79‐year‐old woman with asthma and idiopathic pulmonary fibrosis was admitted for a first episode right apical secondary spontaneous pneumothorax. Due to a lack of pleural separation inside the safety triangle, Rocket® Pleural Vent™ was inserted in the second intercostal space, mid‐clavicular line, as guided by thoracic ultrasound. Although the lung fully re‐expanded the next day, there was persistent air leak and so chemical pleurodesis with oxytetracycline was performed with success. There was no recurrence of pneumothorax and the patient was discharged two days afterwards, and a follow‐up X‐ray taken eight months later did not reveal any recurrence of the right‐sided pneumothorax. This is the first reported case of successful chemical pleurodesis through Rocket® Pleural Vent™ for pneumothorax, which may serve to provide additional alternatives to the management of secondary spontaneous pneumothorax that is indicated for pleurodesis after lung re‐expansion.

## Introduction

We report the first successful case of chemical pleurodesis with oxytetracycline through Rocket® Pleural Vent™ for secondary spontaneous pneumothorax (SSP).

## Case Report

**Figure 1:**
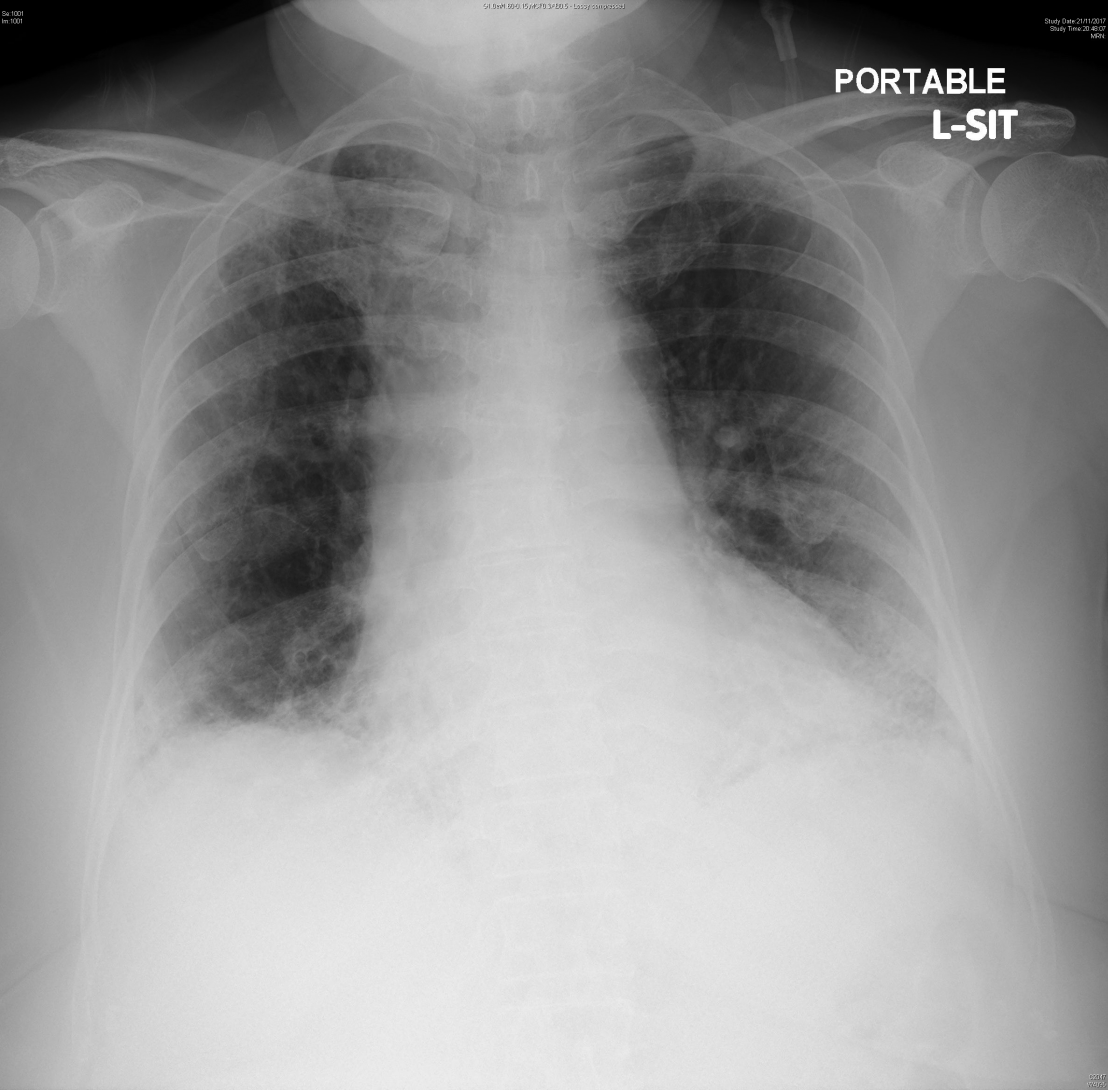
Chest radiograph on admission showing 1.3 cm right apical pneumothorax. Interstitial thickening, consistent with her history of idiopathic pulmonary fibrosis, is also evident especially in bilateral lower zones.

**Figure 2:**
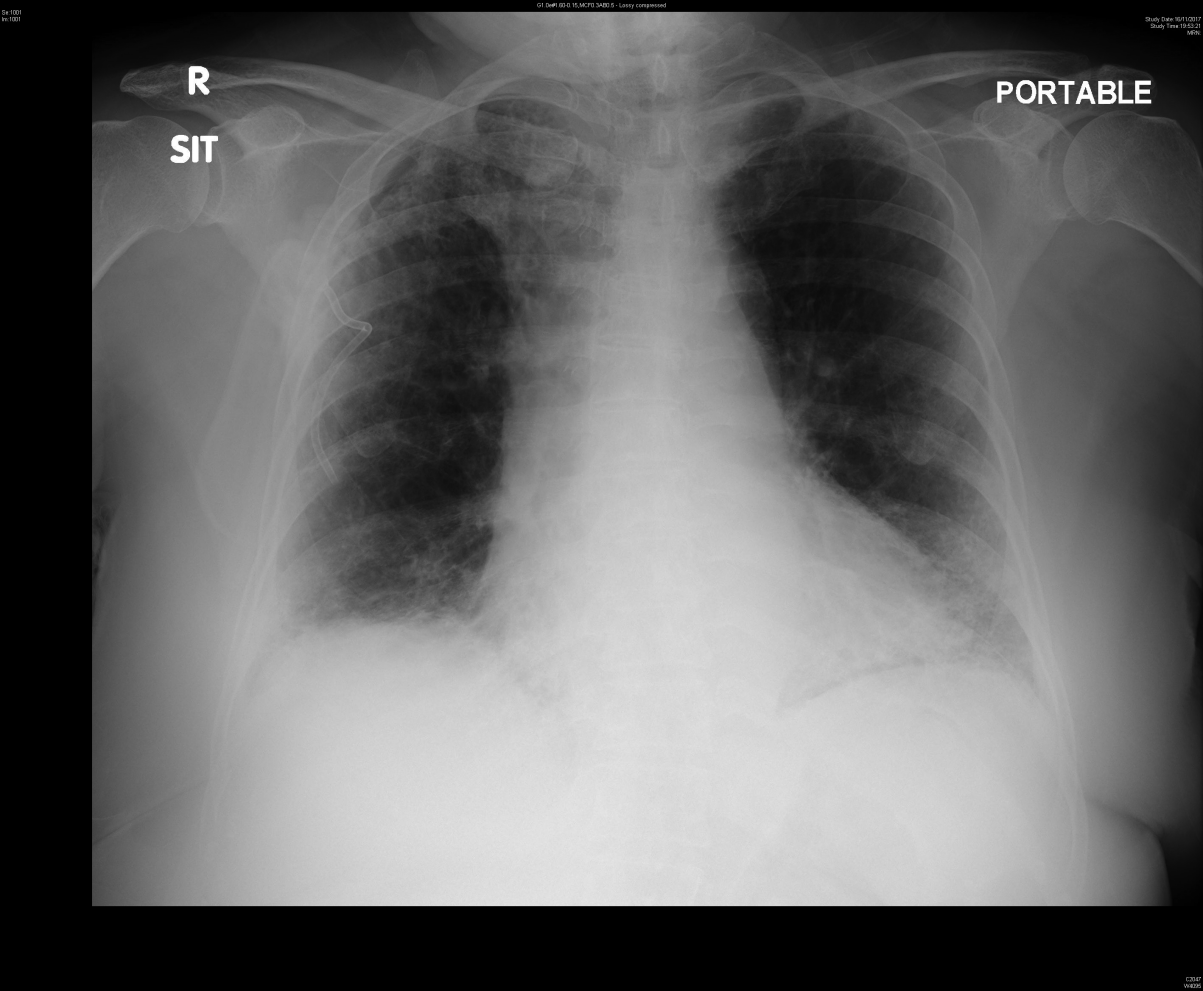
Chest radiograph after oxytetracycline pleurodesis with Rocket® Pleural Vent™ removed, showing full lung re‐expansion.

**Figure 3:**
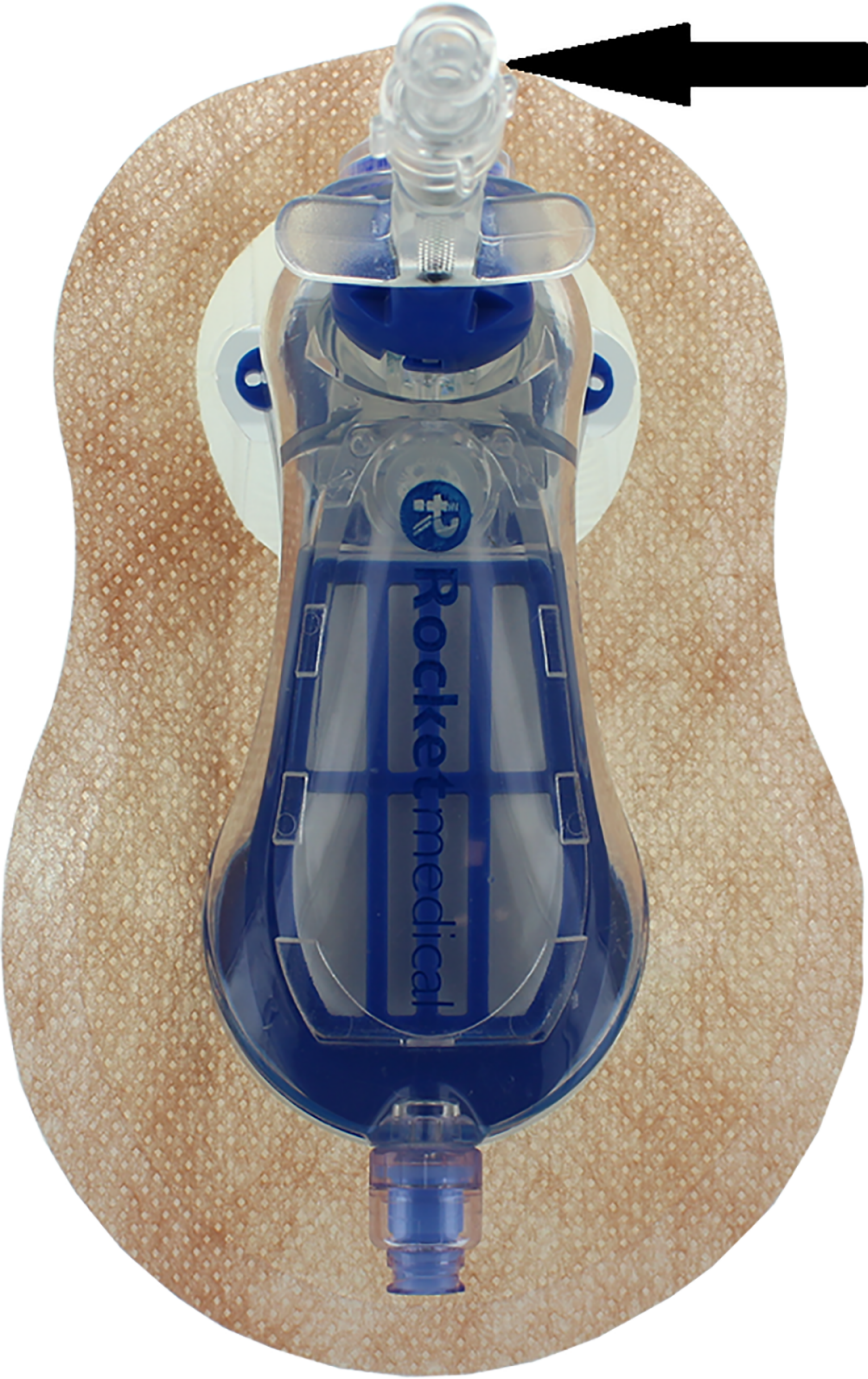
Rocket® Pleural Vent™ device with the injection port shown by the arrow.

A 79‐year‐old woman, with a history of asthma and idiopathic pulmonary fibrosis, presented with sudden‐onset dyspnoea, and was diagnosed as a first episode of right apical SSP. The lung function test before admission showed a forced expiratory volume in one second of 1.44 L (87% predicted) and a forced vital capacity of 1.76 L (76% predicted). Chest radiography on admission showed 1.3 cm right apical pneumothorax (Fig. 1). The pneumothorax did not resolve with conservative management and the patient remained symptomatic. Bedside ultrasonography confirmed a lack of pleural separation inside the safety triangle. Because the patient was fearful of conventional argyle intercostal drain insertion via the second intercostal space approach, Rocket® Pleural Vent™ was inserted instead. The lung fully re‐expanded the next day (Fig. 2), but when we connected the Pleural Vent™ to an underwater seal system prior to its planned removal, persistent and continuous air leak was observed. The patient was reluctant for surgical pleurodesis; therefore, chemical pleurodesis with oxytetracycline was subsequently performed. The chemical pleurodesis was successful, and there were no adverse events from the procedure. After capping off the Pleural Vent™ to confirm there was no recurrence the day after the pleurodesis, the device was removed uneventfully (Fig. 3). There was no recurrence of pneumothorax and the patient was discharged two days after chemical pleurodesis, and a follow‐up X‐ray taken eight months later did not reveal any recurrence of the right‐sided pneumothorax.

## Discussion

Compared with primary pneumothorax, SSP has a higher chance of persistent air leakage and progression; hence, pleural drainage is often necessary 1, 2. The reported rate of recurrence is up to 47% 3. Insertion of conventional intercostal drain can be difficult in cases of loculated or apical pneumothorax, and the procedure can cause significant pain and discomfort. As the intercostal drain needs to be connected to underwater seal, ambulation is heavily impaired, and it can lead to physical deconditioning. In SSP patient with a drain in situ, pleurodesis should be seriously considered to minimize the chance of recurrence, especially for patients with underlying condition that is known to worsen slowly over time (like the interstitial lung disease in our patient) 1, 3. Talc and tetracycline or its variants are frequently used as sclerosant for chemical pleurodesis especially since these patients are often poor surgical candidates 3.

Rocket® Pleural Vent™ is a thoracic decompression device with an 8‐Fr pigtail catheter mounted together with a heimlich valve in a T‐shape configuration designed for the treatment of spontaneous, iatrogenic, or traumatic simple pneumothorax 4. The advantage of this device is that it only requires a small incision and is a single‐pass insertion procedure, and it allows for full patient mobility during treatment as continuous connection to underwater seal is not necessary. From our experience, chemical pleurodesis with oxytetracycline through the Rocket® Pleural Vent™ appears to be a safe and feasible option for patients with pneumothorax while providing the additional benefit of early, near full ambulation to prevent deconditioning, which is not uncommon among patients with chronic respiratory diseases. Figure 3 illustrated the Rocket® Pleural Vent™ and the injection port (arrow).

Due to the small calibre of catheter, we are uncertain whether the use of talc (which is known to be a more effective sclerosant) might be feasible. In the rare event of acute complications with talc pleurodesis, there may be concern that rapid drainage of sclerosant‐containing mixture will be more difficult with this device, but this is likely due to the positioning rather than the instrument itself, and would have been the same for any standard drain/pigtail catheter via the second intercostal space approach. The avoidance of talc as a sclerosant might also lower this risk to a certain degree.

This is the first reported case of successful case of chemical pleurodesis through Rocket® Pleural Vent™ for pneumothorax. It is hoped that this report may serve to provide evidence of an alternative to the management of SSP that is indicated for pleurodesis after lung re‐expansion.

In conclusion, chemical pleurodesis with oxytetracycline through Rocket® Pleural Vent™ is a safe and feasible option for pneumothorax. It can be considered as an option for management of secondary pneumothorax which requires chemical pleurodesis.

### Disclosure Statement

Appropriate written informed consent was obtained for publication of this case report and accompanying images.
